# GenMAPP 2: new features and resources for pathway analysis

**DOI:** 10.1186/1471-2105-8-217

**Published:** 2007-06-24

**Authors:** Nathan Salomonis, Kristina Hanspers, Alexander C Zambon, Karen Vranizan, Steven C Lawlor, Kam D Dahlquist, Scott W Doniger, Josh Stuart, Bruce R Conklin, Alexander R Pico

**Affiliations:** 1 Gladstone Institute of Cardiovascular Disease, 1650 Owens Street, San Francisco, CA 94158 USA; 2 Pharmaceutical Sciences and Pharmacogenomics Graduate Program, University of California, 513 Parnassus Avenue, San Francisco, CA 94143, USA; 3 Functional Genomics Laboratory, University of California, Berkeley, CA 94720 USA; 4 Department of Biology, Loyola Marymount University, 1 LMU Drive, MS 8220, Los Angeles, CA 90045 USA; 5 Computational Biology Graduate Program, Washington University School of Medicine, St. Louis, MO 63108 USA; 6 Department of Biomolecular Engineering, University of California, Santa Cruz, CA 95064 USA; 7 Department of Medicine, University of California, San Francisco, CA 94143 USA; 8 Department of Molecular and Cellular Pharmacology, University of California, San Francisco, CA 94143 USA

## Abstract

**Background:**

Microarray technologies have evolved rapidly, enabling biologists to quantify genome-wide levels of gene expression, alternative splicing, and sequence variations for a variety of species. Analyzing and displaying these data present a significant challenge. Pathway-based approaches for analyzing microarray data have proven useful for presenting data and for generating testable hypotheses.

**Results:**

To address the growing needs of the microarray community we have released version 2 of Gene Map Annotator and Pathway Profiler (GenMAPP), a new GenMAPP database schema, and integrated resources for pathway analysis. We have redesigned the GenMAPP database to support multiple gene annotations and species as well as custom species database creation for a potentially unlimited number of species. We have expanded our pathway resources by utilizing homology information to translate pathway content between species and extending existing pathways with data derived from conserved protein interactions and coexpression. We have implemented a new mode of data visualization to support analysis of complex data, including time-course, single nucleotide polymorphism (SNP), and splicing. GenMAPP version 2 also offers innovative ways to display and share data by incorporating HTML export of analyses for entire sets of pathways as organized web pages.

**Conclusion:**

GenMAPP version 2 provides a means to rapidly interrogate complex experimental data for pathway-level changes in a diverse range of organisms.

## Background

Advances in DNA microarrays, RNA interference, and genome-wide gene engineering have contributed a wealth of genomic data to the public domain. The average researcher is faced with the challenge of connecting these genome level results to specific biological processes. Therefore intuitive tools for integrating, analyzing, and displaying this data are welcomed by many biologists. One popular approach is pathway-oriented data analysis, which enables biologists to interpret genomic data in the framework of biological processes and systems, rather than in a traditional gene-centric manner.

We developed Gene Map Annotator and Pathway Profiler (GenMAPP) as a free, open-source, stand-alone computer program for organizing, analyzing, and sharing genome-scale data in the context of biological pathways [1]. GenMAPP was initially released in 2001 and has been widely used with over 15,000 unique user registrations and over 250 publications citing its use. GenMAPP allows users to view and analyze genome-scale data, such as microarray data, on biological pathways, Gene Ontology terms or any other desired grouping of genes. These groupings are represented and stored in GenMAPP as "MAPPs". GenMAPP automatically and dynamically colors genes on MAPPs according to data and criteria supplied by the user. In addition, GenMAPP allows investigators to easily access annotation for genes at major genomic databases, such as Ensembl [2], Entrez Gene [3], and Gene Ontology (GO) [4]. Using the integrated MAPPFinder tool, researchers can rapidly explore their data in the context of pathways and the GO hierarchy by over-representation analysis [5].

GenMAPP was developed by biologists and remains focused on pathway visualization for bench biologists, our major user base as judged from publications citing GenMAPP. Unlike other computational systems biology tools (e.g., BioSPICE [6], CellDesigner [7], E-Cell [8]), GenMAPP is not designed for cell/systems modeling. GenMAPP focuses on the immediate needs of bench biologists by enabling them to rapidly interpret genomic data with an intuitive, easy-to-use interface.

## Implementation

GenMAPP is implemented in Visual Basic 6.0 and is available as a stand-alone application for Windows operating systems [1]. The program includes an automatic update feature that allows rapid and reliable updates to the program and documentation.

The three main data components in GenMAPP – experimental data (.gex), gene databases (.gdb), and pathways (.mapp) – are stored in separate files accessible by GenMAPP. All three file types are stored in Microsoft Jet format. Experimental datasets store any data imported by the user, together with a set of custom coloring criteria (color sets). The gene databases contain species-specific gene annotation from a number of public resources. Databases are created through an ETL (Extract, Transform, and Load) process, by which information is collected from Ensembl, Entrez Gene, Affymetrix [9], and GOA (UniProt) [10] and reassembled. Annotations supported by GenMAPP include Ensembl gene IDs, UniProt IDs, Entrez Gene IDs, Gene Symbols, UniGene IDs, RefSeq protein IDs, HUGO IDs, GO terms, Affymetrix probe set IDs, RGD IDs (rat), MGI IDs (mouse), SGD IDs (yeast), FlyBase IDs (fruit fly), WormBase IDs (worm), ZFIN IDs (zebrafish), InterPro IDs, EMBL IDs, PDB IDs, OMIM disease associations, and Pfam IDs. MAPPs contain a set of gene or protein identifiers as well as optional graphical elements which are laid out manually. It is up to the author of the MAPP to choose how to illustrate activation, inhibition, compartments, etc. There is no graph underlying MAPPs, there are no formal nodes and edges: the gene boxes are data-linked, but all lines, edges and sub-groupings are illustrations only. Each MAPP can also contain a record of the author and any relevant literature references. GenMAPP does not restrict users to particular semantics. A MAPP can represent any gene set whether it is a metabolic pathway, a signaling pathway, a disease process or an arbitrary set. The pathway archives GenMAPP distributes undergo general review and revision by the GenMAPP staff.

Databases and pathway archives are available through the Data Acquisition Tool in GenMAPP and from the GenMAPP website. The tools known as MAPPFinder 2 and MAPPBuilder 2 are bundled with and accessible from GenMAPP. MAPPBuilder creates .mapp files from imported lists of genes, and MAPPFinder [5] computes permutation test P values for over-representation of differentially expressed genes in individual GO categories and MAPPs. Westfall-Young adjusted P values [11] are included as a control for multiple testing.

## Results and discussion

GenMAPP version 2 provides 1) new built-in features to support user data import and mapping, 2) expanded pathway resources and 3) increased support for different high-throughput biological assays. These improvements substantially increase the usability and flexibility of this tool for pathway level genomic analysis.

### GenMAPP version 2 new features

Several new features have been implemented in GenMAPP version 2. A new gene database schema supports a variety of gene and protein identifiers, annotations, and microarray probe set IDs, more thoroughly connecting user data to the archive of pathway MAPPs and Gene Ontology terms and to external gene annotation. A new visualization mode allows for simultaneous access to multiple data points, statistics or custom annotations. A new export option packages sets of pathways, including data, to a web-ready format for display and browsing.

### Expanded gene and species support in GenMAPP version 2

A major shortcoming of GenMAPP 1.0 and other pathway analysis programs has been the limited number of species supported, permitting analysis of a few model organisms (human, mouse, rat, and yeast) and a few gene identifier or ID systems (GenBank, SGD, and UniProt). To solve this problem, the GenMAPP version 2 gene database schema has been redesigned to allow expanded gene content and greater species support. Support of many diverse gene and protein ID systems is essential to establish critical relationships between disparate sources of information, providing greater flexibility for users importing data associated with virtually any identifier. In addition to expanded gene and protein ID support, secondary annotation systems such as GO, OMIM, and PDB have been added into the GenMAPP gene databases. These IDs and annotations are provided on HTML "backpages" of MAPP gene objects, providing critical links to primary resources. As additional genomes are assembled and annotated, GenMAPP can readily integrate the information and support pathway analysis for these species.

Databases in GenMAPP version 2 are created through a semi-automated process, using information extracted from major public resources, primarily Ensembl, Entrez Gene, UniProt, and Affymetrix. The process of extracting gene information has been greatly simplified by populating our gene database with data from Ensembl's "mart" tables [12], which effectively integrates gene information for major sequenced genomes. GenMAPP.org currently distributes databases for eleven species: human, mouse, rat, yeast, worm, zebrafish, fruit fly, mosquito, chicken, dog, and cow. GenMAPP version 2 also supports user-defined additions to these databases as well as the creation of custom gene databases for any other species. The ability to create custom databases is of vital importance to research groups working with model organisms not supported by the major public databases. This feature is supported by only one other pathway analysis tool we are aware of [13]. Creating a custom database is a collaborative effort where GenMAPP developers generate a template database containing relevant GO term associations for the species of interest. A user interface within GenMAPP version 2 allows users to add to the template database by importing additional gene and annotation information as a set of relational tables. The build process can be completed entirely using GenMAPP and common spreadsheet programs (e.g., Excel), without the need for specialized database software. The resulting database has full GenMAPP functionality, including the ability to display information on HTML backpages, link to external sources, and perform global GO queries using MAPPFinder. Custom GenMAPP version 2 databases are currently available for *Escherichia coli *K12 (KDD and John David N. Dionisio, personal communication) and *Saccharomyces pombe *[14]. A detailed manual describing the process of creating a custom gene database is available at GenMAPP.org.

### Visualizing complex genomic data

As microarray experimental designs grow increasingly complex researchers require tools to examine data across multiple time-points and conditions, and over multiple datasets. The types of biological entities measured have also increased. Various array platforms measure polymorphisms, splice variants, regulatory protein binding and genomic amplifications and deletions. Methods for visualizing the complex outputs from these technologies have not been well established and remain a critical challenge for researchers. With previous versions of GenMAPP, users could view multiple sets of criteria only serially. For example, genes up-regulated at different time-points of an experiment could be viewed by creating a custom set of coloring criteria (color set) for each time point. While informative, this method is not well suited to assess the temporal effects of gene level changes over an extended time or to examine multiple data simultaneously. To expedite the analysis of such datasets, GenMAPP version 2 now allows multiple color sets to be viewed simultaneously, depicted as vertical stripes within each gene box. In the case of multiple time points, the stripes could represent the criteria at each time point.

**Figure 5:**
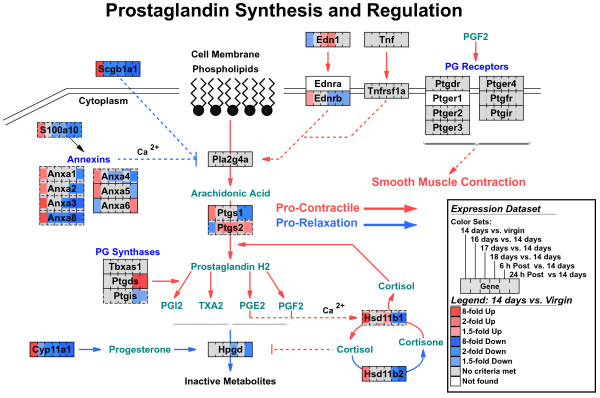
Striped view of multiple time-point comparisons. Gene expression data from mouse uterine smooth muscle from mid-to-late pregnancy through postpartum are shown as striped color sets on a pathway for prostaglandin synthesis and regulation. For each comparison, a separate color set was generated, with eight colors designating different fold thresholds. The order of the criteria dictates the priority for how a gene box is colored. For this dataset, the striped view allows comparison of expression changes that are predicted to promote versus block contraction during distinct phases of pregnancy. Multiple probe sets from the microarray linking to a gene are indicated by a dashed edge around the gene box. The central color of the gene box corresponds to the predominant criterion met (mode) and the rim colors represent the second most prevalent criterion met.

The ability to view multiple color sets concurrently can also be extended to datasets where different biological substrates are examined, such as transcription and mRNA splicing. Demand for this feature is increasing because current microarrays can assay distinct regions of mRNA transcripts, such as exons and exon junctions, thereby allowing assessment of both transcriptional changes and changes in splice isoform expression. While there are many possible ways to view such data, using multiple color sets in GenMAPP is now a powerful way to explore such complex data in a single view. Similar visualization options are only available in a few freely available [15] and commercial applications [16,17].

### Batch export of data to the web

In addition to visualizing data on pathway MAPPs, GenMAPP version 2 also exports pathways with data to various graphical formats and to the web. Because genome-scale data are difficult to share with a larger community, GenMAPP version 2 includes the option to export any number of MAPPs with their associated data to an organized web-ready format. This MAPP Set Export feature allows any or all established color sets to be exported with the pathway, including the striped view of multiple color sets. Instead of static images, each MAPP retains its interactive features, such as gene backpage information, including data display, gene annotations, and hyperlinks to external resources. The different criteria can be browsed through a pull-down tab on each exported MAPP. The MAPP Set can be navigated through an index of all MAPPs or through a gene index, which stores all gene-to-MAPP relationships for all related gene/protein IDs. MAPP Sets are stored in HTML format, ready for immediately posting on any web site, where collaborators can browse the data independently of the GenMAPP program. An example of how a GenMAPP MAPP Set can be used to display large-scale data is the International Gene Trap Consortium web site [18], where thousands of publicly available gene trap ES cell lines can be viewed in the context of biological pathways [19]. This method of data presentation allows users to quickly share information over the Internet and perform efficient searches for gene pathway information. Batch export of fully interactive pathways and user data is not available in other pathway analysis tools we are aware of.

### New Pathway Resources

Integral to any pathway analysis tool is its access to pathway content. One of the goals of the GenMAPP project is to facilitate community curation of pathway content. GenMAPP's built-in drawing tool allows users to illustrate biology and associate gene objects with identifiers maintained in a given gene database. The ability to customize the layout and to annotate a pathway with basic graphics provides a powerful means of communication to the biological community. The expertise of the biological research community is the most important source of new pathway information, and GenMAPP's pathway content is primarily contributed by this community. We have added several new sources of MAPPs. For example the NetPath project is a human pathway annotation project, initiated by the Pandey lab at Johns Hopkins University [20,21] and the Institute of Bioinformatics [22]. The NetPath group has produced 10 cancer and 10 immune pathways in GenMAPP, BioPAX [23], and PSI-MI [24] formats, and are planning a substantial increase within the first year. Another ongoing pathway curation effort is being performed by undergraduate research students directed by Dr. Kam Dahlquist. These students have contributed 120 yeast pathways that were created by hand using the SGD BioCyc metabolic pathways [25] as templates. The GenMAPP pathway archives also include selected content from KEGG [26], Reactome [27,28], The European Nutrigenomics Organization [29], Neurocrine Biosciences, PharmGKB [30], and various academic laboratories. The content from these resources was manually migrated by the MAPP authors with the exception of the "KEGG Converted" archive, which is not updated or synchronized. The pathways from community resources are collected and organized at GenMAPP.org and automatically downloadable through the GenMAPP program.

We now also provide pathways that have been mapped through homology so that users with genomic data from relatively unsupported species can perform pathway analyses. These homology MAPPs represent a starting point for further curation, an interim solution until species-specific pathways are elucidated and contributed. Another means of increasing the biological content available to the user is the extension of existing pathways using interaction and coexpression data. Together, these methods only begin to address the paucity of pathway content available for the analysis of complex genomics data across the multitude of organisms.

### Making homology MAPPs

**Figure 1:**
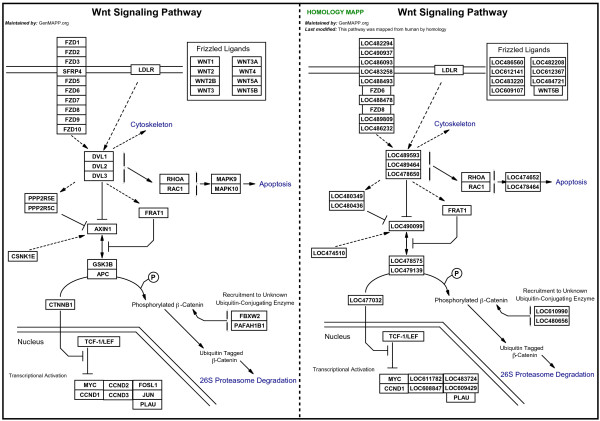
The WNT-signaling pathway is shown in GenMAPP for human and dog (left to right). The dog pathway MAPP was mapped from the original curated human pathway MAPP by using homology information from Homologene and Ensembl. Additional information in the top-left corner of the MAPP indicates the origin.

Despite the relative ease with which we can gather gene information for many species, pathway information is generally not available for many of the newly supported species (Table 1). To address this problem, we implemented a strategy that utilizes the existing pathway content in our pathway archives. Using publicly available gene homology information [12,31], we generated pathways for several species from our archive of existing human pathways (Figure 1).

**Table 1:** Number of GenMAPP MAPPs for GenMAPP supported species

**Species**	**Contributed**	**Homology**	**KEGG Converted**
Human	109		
Mouse	109		
Rat	100		
Dog		94	
Cow		87	
Chicken		85	
Zebrafish		19	18
Fruit fly	2	25	89
Worm		19	87
Yeast	122*	9	

Column Headers: *Contributed*: MAPPs contributed to the GenMAPP project. *Homology*: MAPPs mapped from human pathways using homology information. *KEGG Converted*: MAPPs automatically created from the KEGG resource. Note: *Includes 120 SGD metabolic MAPPs contributed by undergraduate research students mentored by Dr. Kam Dahlquist.

The process of rapidly mapping pathways between species relies on the Converter function in GenMAPP version 2, which allows for conversion of genes on MAPPs between gene ID systems in the database without altering the graphical layout of the MAPP. MAPP conversion is possible between any gene ID systems linked in the database; adding homology information to a GenMAPP database consequently enables conversion of MAPPs between species.

**Figure 2:**
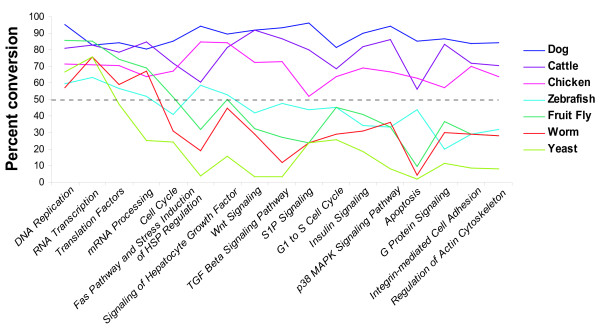
Conversion rate (percentage of genes converted) for MAPPs in the Cellular Process category of the Contributed archives. Colored lines indicate conversions per species; dashed line indicates the 50% cutoff for inclusion in the Homology MAPPs archive. As expected, highly conserved processes showed high conversion rates across species (far left), while more specialized processes were homologous only among more closely related species.

The GenMAPP human MAPPs were chosen as template MAPPs because they represent the highest quality of curation in our archive. Homology information between human and the applicable target species was obtained from Homologene [31] and from EnsMart [22] (for cow only)(Algorithm details in Supplemental Data). For simplicity we restricted the use of data from these resources to 1:1 gene relationships between template and target species. This restriction reduces clutter in the converted MAPPs and avoids potentially ambiguous homology relationships. Conversion rates (percentage of genes converted) were calculated for each pathway MAPP (Figure 2 and Supplemental Data). To maintain reasonable gene content on MAPPs, a cutoff of 50% for the conversion rate was set for inclusion in the MAPP archives. The cutoff of 50% was chosen based on the qualitative assessment of structure and pathway information retained following conversion (see supplemental data for MAPP conversion rates). Qualitatively, the conversion rates correlated with the expected conservation of biological processes across species. The MAPPs representing the central dogma of DNA replication, RNA transcription and translation, for example, were converted with high fidelity from human to each of the target species, whereas specialized signaling pathways failed to be translated beyond dog, cow and chicken. This strategy of utilizing public homology information, existing pathway information and the Converter function can be applied to any species with available homology information to a species for which pathway information exists. Instructions for translating MAPPs between species using the GenMAPP Converter is available at GenMAPP.org [32].

The development of homology MAPPs in GenMAPP builds upon similar efforts at other databases [27] and addresses the dearth of pathway content that can be queried computationally. However, it is important to note that these MAPPs are not genuine species-specific pathways, but rather translations of human pathways where target species genes have been mapped based on homology. This distinction is important since accurate pathway inference requires knowledge that the particular biological process and molecular interactions are conserved between organisms and that predicted homologues encode for gene products that perform the same biological function. Another current limitation is that, unlike several other resources [27,33,34], the reactions in a GenMAPP pathway are illustrations rather than computable networks that allow for identification of conserved interactions. Furthermore, pathways for non-mammalian species are mapped from human rather than the most closely related organism. As such, these homology MAPPs are by no means equal to the quality of manually curated MAPPs. For that reason homology MAPPs are distributed as a separate archive, accompanied by a README file explaining the nature of these MAPPs. They nonetheless offer an immediate and concrete solution for many researchers studying organisms with minimally annotated genomes not supported by other analysis programs. It is our hope that these pathways will serve to nucleate additional curated pathways. Furthermore, the information provided by pathway representations of known biology, especially for minimally annotated genomes, is crucial not only for analyzing large-scale datasets, but also for assigning gene function.

### Extending pathways

**Figure 3:**
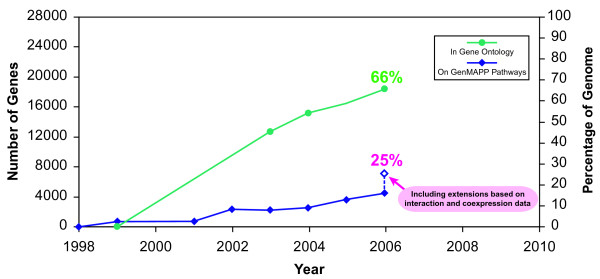
Number of mouse genes represented on GenMAPP Pathways and in Gene Ontology. The unique gene content in the GenMAPP pathway archive is traced over time (blue) for the mouse genome in terms of the number of genes (left axis) and corresponding percentage of the genome (right axis). For comparison, the unique gene content annotated by Gene Ontology is shown (green). Significant gains in absolute gene content were made by collecting new pathways targeting new biology (e.g., NetPath) and by extending pathways with orthogonal datasets from coexpression and protein-protein interaction networks (latest GenMAPP count at 7095 genes, or ~25% of the genome).

The use of homology mapping addresses the critical need to extend biological representations across species. Yet it is also necessary to expand the pathway content within a given species. In the case of mammalian model organisms, such as mouse, only ~14% of annotated genes in the genome are represented in curated pathways (from the combined archives of GenMAPP, KEGG, BioCarta [35] and Reactome). Figure 3 illustrates the collection of curated pathways in the GenMAPP archive over time, which, in terms of gene content is >90% redundant with BioCarta and Reactome. The collection of curated pathways from the scientific community is a slow, iterative process that requires the synthesis of a variety of evidence. Such evidence is being cataloged in numerous databases as protein-protein interactions, genetic interactions, and coexpression patterns, which are rapidly expanding with the advent of large-scale, high-throughput assays. But it remains a challenge to form meaningful networks from this data and grow our understanding of pathways.

**Figure 4:**
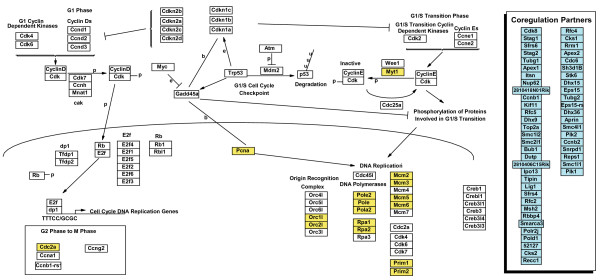
G1 to S cell-cycle control pathway extended with genes from a coexpression network. All genes assigned to the original pathway were queried against the coexpression network. Yellow designates the genes found in the coexpression network and blue designates their coexpression partners that were extracted from the network and added to the pathway.

To address this challenge, we created a new pathway resource, which incorporates additional genes into our existing set of pathways using prior evidence. This method of pathway extension has been previously used to include new genes predicted to expand and enhance the content of existing pathways and gene sets [36]. The method can work with any type of data that can be modeled as pairs of linked genes. The most obvious example is protein-protein interactions, where the link between genes represents the physical association of two proteins. The link could also represent coexpression, transcriptional regulation, or literature search results. The extension method is currently implemented as a set of in-house Perl scripts used as an accessory to GenMAPP to expand a given pathway. Each pathway MAPP is processed individually. First, the gene IDs are extracted from the pathway and converted to a uniform ID system (e.g., Entrez Gene). The resulting gene list is used to query one or more specified databases (e.g., protein-protein interactions). A threshold is set for including new genes (e.g., one or more links to original gene list). Finally, the new genes are added to a side panel of the original MAPP, separate from the curated pathway, and the interaction partners are noted and stored in the remarks field of each involved gene (Figure 4).

Using this approach, we extended the GenMAPP curated pathway archives for mouse with two types of data: protein-protein interactions and coexpression data [37] (see supplemental data). The coexpression links were derived from a network analysis of correlated gene expression across multiple species networks [37] under the premise that genes that maintain an evolutionary conservation of coregulation often participate in a related biological process [38,39]. With the additional genes added from these datasets, we have significantly increased the coverage (~25%) per genome (Figure 3). It is important to distinguish the added genes from those originally in the pathway since the added genes are not necessarily *involved *in the pathway; rather, they are *related *to the pathway by a particular type of evidence. Having access to this related information in the same view as the pathway allows for simultaneous data visualization and statistical analysis using MAPPFinder. These extended pathways may also serve as launching points for improved pathway curation by the community and as a predictive method for identifying new pathway interactions.

### Examples of pathway analysis

Here we explore three of the many examples of how GenMAPP version 2 can be used to analyze data from complex genomic experiments and the types of biological insights potentially gained.

### Gene expression time course analysis

In figure 5, we display gene expression data from multiple time-point comparisons for the myometrium during gestation [40]. There are two baselines in this analysis: virgin non-pregnant (NP) myometrium and mid-pregnancy myometrium. The comparison allows the user to simultaneously examine the effects of pregnancy as compared to non-pregnant animals and the specific temporal effects leading up to labor through postpartum.

**Figure 6:**
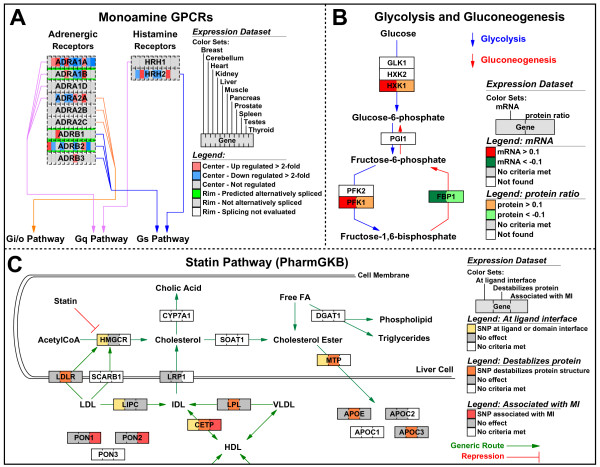
Striped view of multiple data types. (A) Transcription and splicing data, collected on whole-genome exon tiling microarrays (see supplemental methods), are represented by stripes of color on a functionally organized list of monoamine G-protein-coupled receptors. Transcriptional changes for 11 different human tissues are displayed as the center color of the gene box, and splicing for the gene across all tissues is displayed as the rim color. (B) In the context of glycolysis and gluconeogenesis, mRNA and protein levels change in response to carbon source perturbation in *Saccharomyces cerevisiae *growing on galactose or ethanol. The color on the left side of the gene box illustrates mRNA changes; the color on the right indicates corresponding protein-level changes. (C) A variety of SNP parameters can be viewed simultaneously using the striped view. SNP distribution (dbSNP ), structure-based functional predictions (LS-SNP ), and myocardial infarction (MI) association data are combined to asses the coverage of the SNP panel over a pathway depicting the role of statin drugs. To view the complete versions of these MAPPs with live backpages see Supplemental Data.

The prostaglandin synthesis and regulation pathway contains molecular interactions that are critical in the transition of the myometrium from a relatively quiescent tissue throughout pregnancy to a highly contractile tissue at term. By viewing multiple time-point comparisons in this pathway, one can easily see which genes are differentially expressed just prior to the onset of labor (18 days of pregnancy) compared to mid-pregnancy (14 days of pregnancy) (e.g. Ptgs2, Edn1 and Hsd11b1) alongside the relative expression of these genes at mid-pregnancy versus the virgin state (first stripe). Making such comparisons in the new version of GenMAPP is relatively straightforward and flexible, supporting not only multiple data points, but also multiple types of data (see SNP example, figure 6c). In GenMAPP version 2, the user can also select any combination of color sets to view on a given MAPP simply by selecting them from the "Choose Color Set" pull-down.

### Analysis of whole-genome exon array data

As the feature size of DNA microarrays have decreased, the number of probes hybridizing to specific targets has increased by well over an order of magnitude. In the example shown in Figure 6a, we examined a publicly available microarray dataset that measured the expression of all known and predicted exons from 11 different adult human tissues [41]. From these data, both gene expression changes between tissues and splicing scores can be calculated for all genes (see supplemental methods). GenMAPP version 2 can display this information in each gene box, with the central color stripes indicating relative expression change for each tissue (red or blue) and the rim color designating a threshold for the significance of an alternative splicing call (green, gray, or white). This strategy takes advantage of how GenMAPP prioritizes assignment of central and rim colors of a gene box based on the order of the underlying data. Viewing related identifiers to a given gene as a secondary rim criterion can provide critical information to the analysis and is a unique feature of GenMAPP. When viewed in the context of Monoamine G-protein coupled receptors, we can clearly identify in which tissues a gene is most highly expressed (bright red center color) and which genes have a significant alternative splicing call (green rim color). By creating a color set for each of the 11 tissues and selecting "all" for visualization, both the tissue specific regulation of gene expression and the likelihood of splicing can be assessed in a single view. The results from this dataset can be exported for any given set of pathways with web-ready images and HTML backpages for each and every gene. The web export function allows researchers to navigate and effectively communicate the impact of both gene expression and splicing on specific pathways and genes (see the GenMAPP website [42] for this example and others).

### Combining proteomic and gene expression data

In another example, gene expression and proteomic data [43] is viewed concurrently as two adjacent stripes of color (Figure 6b). The example displays data from an experiment measuring both mRNA and protein levels in yeast in response to changes in carbon source. Simultaneously visualizing changes at the transcript and protein level in the context of pathways represents a more informative depiction of the system-level changes occurring in the organism than if either data was analyzed alone. The flexibility of combining any number of disparate data types in a single view is a relatively uncommon feature in pathway analysis tools. To view two data types side by side, datasets are combined into a single spreadsheet before import into GenMAPP. There are no restrictions on the nature of data that can be viewed as independent, adjacent color sets, provided that the data links to the GenMAPP gene database.

### Integrating genomic, phenotypic and structural information for polymorphism data

One of the key principles of pathway analysis is the integration of multiple pieces of information in order to assess new data in the context of known biology. In studying polymorphic, or SNP, differences that may contribute to disease, the ability to compare the distribution of polymorphisms in the population along with phenotypic and protein product effects in the context of biological pathways provides both a birds-eye view and detailed dissection of how specific changes might impact larger biological systems. An example of how these different types of biological data can be combined is shown in Figure 6c using data from a whole-genome myocardial infarction SNP array experiment [44]. Displaying data in this format highlights genes evidenced by association, experimental and bioinformatics predictions (e.g. CETP, MTP) as well as their relationship to each other and with other genes upstream and downstream of these components. Display formats such as this allow access to multiple modes of gene regulation from a single display.

Although these examples illustrate three possible methods for displaying complex results, users can customize such views and apply them to any combination of data types that have been merged and ordered before import to GenMAPP. This feature provides a means to assess multiple modes of gene regulation and thus new avenues of insight into complex biological relationships.

### Ongoing development of GenMAPP

GenMAPP version 2 provides new tools for analyzing complex data in the context of biological pathways for a variety of genomes. Although the new features of GenMAPP version 2 are a useful starting point for the analysis of complex microarray data, there are still a number of obstacles to overcome. These obstacles include providing cross-platform tools for integrating pathway resources, representing gene features (such as SNPs and splicing variation), and supporting structured pathway vocabularies for more efficient pathway migration, update, curation and exchange.

To accelerate development and take full advantage of the growing base of open source pathway tools we are actively working with the Cytoscape Consortium [45,46] and BioPAX [23] developers to implement GenMAPP-style visualization and analysis methods in a new software framework. The primary aims are (1) to transition to a platform-independent Java code base that is readily integrated with online resources, (2) to support dynamically generated gene databases that not only organize identifiers and aliases, but also sub-gene entities such as transcripts, exons, and polymorphisms, and (3) to provide innovative analysis tools to preprocesses high-throughput datasets preparing them for integration with gene databases and statistical analyses, as well as for abstracted visualization at multiple levels of resolution. We are also working on an XML-based pathway data format that captures relationships, coordinates, and annotations, as well as a Web tool that facilitates pathway content migration, and curation from the community. We anticipate that open source bioinformatics tools such as GenMAPP and Cytoscape will provide researchers with a new view of biology that integrates genomic data with our growing knowledgebase of pathways.

## Conclusion

GenMAPP version 2 represents a step towards fostering the critical link between the biologist and their data, providing powerful analyses and intuitive representations of increasingly large and complex high-throughput datasets.

## Availability and requirements

*Project Name*: GenMAPP

*Project Home Page*: 

*Operating System*: Windows

*Programming Language*: Visual Basic

*Requirements*: Species-specific databases and pathway file collections distributed by GenMAPP.org

*License*: Open-source (Apache)

*Any Restrictions to Use by Non-academics*: None

## Authors' contributions

GenMAPP version 2 features were conceived by BC, KD, AP, NS, SD, KH, KV, AZ and SL. Computer code for the GenMAPP version 2 application was written by SL and SD. AP, NS and KH drafted the manuscript. The pathway extension method was performed by AP and JS; and homology mapping was performed by KH. All authors read and approved the final version of the manuscript.

## Supplementary Material

Additional File 1 Supplemental Methods. Click here for file
