# Supplementary material for: GenMAPP 2: new features and resources for pathway analysis
Source: BMC Bioinformatics. 2007 Jun 24;8:217. doi: 10.1186/1471-2105-8-217 (PMC1924866; doi:10.1186/1471-2105-8-217)
Supplement: Additional File 1 — Supplemental Methods. [file 1471-2105-8-217-S1.doc]

**Supplemental Methods**

### Creating homology MAPPs from human pathways

The rate of conversion for each pathway MAPP was calculated as the percentage of genes converted to the target species compared to the total number of genes on the pathway. After conversion, MAPPs were altered to include the appropriate species-specific gene symbol as the gene name, as well as to remove gene IDs from non-converted genes using an in house Perl script (contact [genmapp@gladstone.ucsf.edu](mailto:genmapp@gladstone.ucsf.edu) for details and access). Homologene and Ensembl algorithms for comparative genomics are described at their respective websites[1, 2]. Conversion rates for 71 MAPPs for 6 different species are provided in a table following References.

***Pathway Extension***

The databases for pathway extension are available for download as tab-delimited files from the GenMAPP website[3, 4]. These include the protein-protein interaction MEGINT database (Meta-Gene Interaction) and COREG (Coexpression Regulation)[5]. The coexpression database can be queried independently to map gene sets across species using the Multi-Species Gene Recommender (MSGR – publication in press)[6]. The protein-protein interactions were collected from multiple species (Hs, Mm, Dm, Ce and Sc) using HPRD [7], Worm Interactome [8], BIND [9], MIPS [10], and DIP [11] and mapped using the best-reciprocal BLASTP [12]. GenMAPP pathway MAPPs were extended with interactions from the listed protein-protein interaction and coexpression databases using a Perl script. For parties interested in obtaining and using the script, please contact: [genmapp@gladstone.ucsf.edu](mailto:genmapp@gladstone.ucsf.edu), as they require a minimal level of programming expertise to setup.

### Analysis of exon tiling data

Microarray .cel files were downloaded from [http://www.affymetrix.com](http://www.affymetrix.com/) for 11 human tissues (33 samples) for approximately 1.4 million sequence features (mainly exon) [13]. Expression values were summarized at the probeset level using the ExACT 1.0 software provided by Affymetrix using quantile normalization and sketch summarization [14]. Associated DABG or “Detection Above BackGround” (a metric for comparing perfect-match probes to the distribution of background probes) p values were generated for all probesets to determine the likelihood of expression. Probesets were aligned to genes and exons based on the genomic coordinates provided for each probeset from the Affymetrix design time annotation files (genome build 35) and from [http://www.ensembl.org](http://www.ensembl.org/). Probesets not aligning to an Ensembl gene encoding genomic loci were excluded from the analysis. The remaining probesets were annotated according to the exon structures provided by Ensembl. Constitutive exons were identified from the Affymetrix annotation files (most over-represented exons in mRNAs or expressed sequence tags). To eliminate nonoptimal hybridization results, if less than 9% of all samples possessed a probesets with a DABG p value <0.001, these probeset were filtered out. Of the remaining probesets, expression levels for those associated with constitutive exons were averaged per gene to obtain a gene expression intensity value. Expression values for nonconstitutive probesets and the summarized gene expression values were used to determine the likelihood of splicing using the MiDAS algorithm [15] through the Affymetrix Power Tools command line application.

To determine the relative expression change (fold) for each tissue, the mean expression value across all tissues was used as a baseline. To visualize the splicing scores and relative tissue expression fold changes in GenMAPP version 2, two rows per gene were stored in the GenMAPP version 2 input text file, the first row containing the gene expression values and the second the splicing scores. GenMAPP version 2 displays the first value encountered as the central gene object color on the MAPP for a given criterion and the second as a rim color (if only two rows for the same gene are found). Therefore, the GenMAPP color criterion for each gene splicing score will automatically be assigned to the rim of that gene on the MAPP. Genes with a relative fold change >2 (up or down) are displayed as the center gene criterion using the multiple criterion display option for all 11 tissues in Figure 6A. To obtain detailed criterion information and raw data, download the associated GenMAPP expression dataset file (GEX) from [http://www.genmapp.org](http://www.genmapp.org/).

### Analysis of combined proteomic and gene expression data

Data were analyzed as described [16]. Data provided as a supplemental table were formatted and imported into GenMAPP with SGD IDs as the primary identifier. Separate Color Sets for transcript-level and protein-level changes were created in GenMAPP. For protein and transcript data, the log_10_ ratio of abundance on galactose vs. ethanol was used for coloring criteria, with a ratio of 0.1 as the cutoff (Figure 6B). The GenMAPP dataset is available for download at http://www.genmapp.org.

# *Pathway analysis of multiple SNP parameters*

Single nucleotide polymorphisms (SNPs) associated with myocardial infarction (MI) were extracted from a table provided by Tobin et al. [17]. MAPPFinder analysis was performed with GenMAPP version 2 to identify the overrepresentation of genes with MI-associated SNPs in the statin pathway (contributed by PharmGKB, www.pharmgkb.org). Additional parameters for the SNPs represented by the genes on this pathway were collected from a public database of structure-based functional SNP predictions (LS-SNP, alto.compbio.ucsf.edu/LS-SNP). Additional parameters included whether the SNP was found near a ligand binding site or domain-domain interface, potentially disrupting binding, and whether the SNP was likely to have a destabilizing effect on protein folding (e.g., by producing a nonconservative amino acid substitution). Additional predictive parameters are available from LS-SNP (not shown). MI-association, ligand/interface location information, and destabilization predictions were combined into a single spreadsheet and imported into GenMAPP version 2 to create a new “Expression Dataset,” using the rs identifiers from dbSNP to link the data to the gene objects on the pathway MAPP. This required using a custom human Gene Database containing a table linking the rs identifier to the primary MAPP ID, in this case, the Ensembl identifier. The resulting simultaneous display of these SNP parameters on the Statin Pathway is shown in Figure 6C.

**References**

### Conversion Rate Table

Performance of homology-based inference of pathway content can be assess by calculated conversion rates. The percentage of genes with 1:1 homology from human to a target species is given for each MAPP in the table. Target species include dog (Cf), cow (Bt), chicken (Gg), zebrafish (Dr), fruit fly (Dm), worm (Ce), and yeast (Sc). Rows are sorted by descending average across all species per MAPP, top-to-bottom. Columns are sorted by descending average across all MAPPs per species, left-to-right.

| **MAPP Name** | **Cf** | **Bt** | **Gg** | **Dr** | **Dm** | **Ce** | **Sc** |
| --- | --- | --- | --- | --- | --- | --- | --- |
| RNA_transcription_Reactome | **62** | **80** | **72** | **65** | **87** | **77** | **77** |
| Krebs-TCA_Cycle | **93** | **87** | **65** | **50** | **75** | **71** | **62** |
| DNA_replication_Reactome | **78** | **81** | **71** | **59** | **85** | **57** | **66** |
| Pentose_Phosphate_Pathway | **87** | **73** | **62** | **62** | **75** | **62** | **75** |
| Ribosomal_Proteins | **72** | **83** | **65** | **54** | **76** | **73** | **62** |
| Synthesis_and_Degradation_of_Ketone_Bodies_KEGG | **80** | **86** | **100** | **60** | **60** | **60** | 20 |
| Mitochondrial_fatty_acid_betaoxidation | **87** | **82** | **75** | **62** | **68** | **68** | 12 |
| Cholesterol_Biosynthesis | **68** | **94** | **87** | 43 | 43 | 18 | **87** |
| Proteasome_Degradation | **74** | **71** | **59** | **54** | **61** | **61** | **58** |
| mRNA_processing_Reactome | **71** | **83** | **65** | **53** | **71** | **65** | 25 |
| Nucleotide_Metabolism | **76** | **71** | **76** | 35 | **58** | 47 | **64** |
| Glycolysis_and_Gluconeogenesis | **86** | **84** | **60** | 48 | **55** | 44 | 48 |
| Heme_Biosynthesis | **88** | 30 | **88** | 44 | **88** | 0 | **77** |
| Glycogen_Metabolism | **83** | **78** | **70** | 35 | 48 | **54** | 45 |
| Translation_Factors | **68** | 38 | **70** | **56** | **74** | **58** | 47 |
| Signaling_of_Hepatocyte_Growth_Factor_Receptor_Biocarta | **76** | **79** | **84** | **52** | **50** | 44 | 15 |
| Acetylcholine_Synthesis | **71** | **71** | **57** | **57** | 42 | **57** | 42 |
| Biogenic_Amine_Synthesis | **72** | **78** | **94** | **50** | **55** | 38 | 0 |
| G1_to_S_cell_cycle_Reactome | **67** | **69** | **68** | **51** | **51** | 31 | 28 |
| Cell_cycle_KEGG | **71** | **72** | **65** | 42 | **53** | 30 | 25 |
| p38_MAPK_signaling_pathway | **80** | **100** | **66** | 33 | 33 | 36 | 8 |
| Oxidative_Stress | **78** | **86** | **64** | 32 | 39 | 39 | 17 |
| Electron_Transport_Chain | **64** | **67** | **64** | 45 | **55** | 40 | 18 |
| MAPK_Cascade | **80** | **73** | **66** | 40 | 36 | 36 | 20 |
| Fas_Pathway_and_Stress_Induction_of_HSP_Regulation_Biocarta | **86** | **60** | **86** | **60** | 33 | 19 | 3 |
| Insulin_Signaling | **78** | **72** | **69** | 34 | 41 | 30 | 18 |
| Wnt_signaling | **72** | **92** | **72** | 41 | 32 | 29 | 3 |
| G13_Signaling_Pathway | **75** | **82** | **72** | 32 | 37 | 32 | 8 |
| Circadian_Exercise | **68** | **77** | **66** | **58** | 31 | 25 | 12 |
| S1P_Signaling | **84** | **82** | **52** | 44 | 24 | 24 | 24 |
| Regulation_of_Actin_Cytoskeleton_KEGG | **70** | **84** | **66** | 33 | 29 | 29 | 8 |
| Monoamine_GPCRs | **79** | **85** | **82** | 17 | 29 | 26 | 0 |
| TGF_Beta_Signaling_Pathway | **86** | **68** | **71** | 47 | 27 | 11 | 3 |
| G_Protein_Signaling | **68** | **83** | **57** | 19 | 36 | 29 | 11 |
| MAPK_signaling_pathway_ KEGG | **75** | **55** | **63** | 40 | 32 | 22 | 12 |
| Calcium_regulation_in_cardiac_cells | **70** | **82** | **60** | 27 | 27 | 25 | 7 |
| Nuclear_receptors_in_lipid_metabolism_and_toxicity | **73** | **82** | **57** | 23 | 21 | 40 | 0 |
| Irinotecan_pathway_PharmGKB | **74** | **81** | 37 | 11 | 33 | 44 | 14 |
| Ovarian_Infertility_Genes | **79** | **62** | **88** | 20 | 20 | 17 | 8 |
| Hypertrophy_model | **75** | 42 | **80** | 30 | 30 | 25 | 10 |
| Eicosanoid_Synthesis | **82** | **64** | **52** | 43 | 21 | 26 | 4 |
| Striated_muscle_contraction | **78** | **80** | **63** | 23 | 21 | 23 | 2 |
| Nuclear_Receptors | **90** | **61** | **58** | 46 | 17 | 14 | 0 |
| HSP70_and_Apoptosis | **78** | **55** | **73** | 26 | 21 | 17 | 8 |
| Adipogenesis_Human | **77** | **78** | **58** | 32 | 17 | 14 | 0 |
| ACE-Inhibitor_pathway_PharmGKB | **70** | 36 | **90** | **50** | 10 | 20 | 0 |
| Peptide_GPCRs | **80** | **74** | **68** | 17 | 18 | 16 | 0 |
| Prostaglandin_synthesis_regulation | **87** | **77** | **64** | 32 | 6 | 6 | 0 |
| Nucleotide_GPCRs | **72** | **88** | **90** | 0 | 9 | 9 | 0 |
| Integrin-mediated_cell_adhesion_KEGG | **75** | 18 | **72** | 30 | 30 | 30 | 8 |
| Steroid_Biosynthesis | **78** | **74** | **75** | 6 | 15 | 3 | 9 |
| Smooth_muscle_contraction | **68** | **55** | **59** | 30 | 24 | 18 | 3 |
| Apoptosis | **74** | **56** | **64** | 45 | 9 | 4 | 2 |
| GPCRDB_Class_C_Metabotropic_glutamate_pheromone | **93** | 49 | **60** | 0 | 26 | 26 | 0 |
| Inflammatory_Response_Pathway | **84** | **82** | 45 | 18 | 9 | 9 | 0 |
| Tryptophane_Metabolism_KEGG_Updated | 45 | **60** | 46 | 24 | 24 | 31 | 13 |
| Urea_cycle_and_metabolism_of_amino_groups_KEGG_Updated | **51** | **54** | 38 | 23 | 35 | 20 | 17 |
| GPCRDB_Class_B_Secretin-like | **66** | **80** | **62** | 12 | 8 | 8 | 0 |
| Small_ligand_GPCRs | **66** | **77** | **61** | 22 | 5 | 0 | 0 |
| Statin_Pathway_PharmGKB | **75** | 8 | **55** | 30 | 25 | 20 | 5 |
| GPCRDB_Class_A_Rhodopsin-like | **67** | **62** | **54** | 11 | 11 | 11 | 0 |
| Blood_Clotting_Cascade | **66** | 41 | **61** | 28 | 19 | 0 | 0 |
| GPCRDB_Other | **52** | **89** | 37 | 8 | 10 | 5 | 0 |
| Matrix_Metalloproteinases | **64** | **71** | 41 | 9 | 6 | 6 | 0 |
| Complement_Activation_Classical | **70** | 24 | **76** | 5 | 0 | 0 | 0 |
| GPCRDB_Class_A_Rhodopsin-like2 | 44 | **63** | 44 | 11 | 0 | 0 | 0 |
| Alanine_and_aspartate_metabolism_KEGG_Updated | 24 | 29 | 21 | 21 | 24 | 19 | 21 |
| Complement_and_Coagulation_Cascades_KEGG | **63** | 19 | **55** | 17 | 3 | 0 | 0 |
| Glucocorticoid_Mineralocorticoid_Metabolism | **81** | 11 | 36 | 18 | 9 | 0 | 0 |
| Gluthation_Metabolism_KEGG_Updated | 32 | 19 | 34 | 13 | 23 | 23 | 11 |
| Cytokines_and_Inflammatory_Response_Biocarta | **82** | 23 | 27 | 15 | 0 | 0 | 0 |
